# Dynamics of COVID-19 progression and the long-term influences of measures on pandemic outcomes

**DOI:** 10.1186/s12982-022-00119-6

**Published:** 2022-12-22

**Authors:** Yihong Lan, Li Yin, Xiaoqin Wang

**Affiliations:** 1 Suntar Research Institute, Singapore, Singapore; 2 Karolinska Institutet, Solna, Sweden; 3 University of Gävle, Gävle, Sweden

## Abstract

The pandemic progression is a dynamic process, in which measures yield outcomes, and outcomes in turn influence subsequent measures and outcomes. Due to the dynamics of pandemic progression, it is challenging to analyse the long-term influence of an individual measure in the sequence on pandemic outcomes. To demonstrate the problem and find solutions, in this article, we study the first wave of the pandemic—probably the most dynamic period—in the Nordic countries and analyse the influences of the Swedish measures relative to the measures adopted by its neighbouring countries on COVID-19 mortality, general mortality, COVID-19 incidence, and unemployment. The design is a longitudinal observational study. The linear regressions based on the Poisson distribution or the binomial distribution are employed for the analysis. To show that analysis can be timely conducted, we use table data available during the first wave. We found that the early Swedish measure had a long-term and significant causal effect on public health outcomes and a certain degree of long-term mitigating causal effect on unemployment during the first wave, where the effect was measured by an increase of these outcomes under the Swedish measures relative to the measures adopted by the other Nordic countries. This information from the first wave has not been provided by available analyses but could have played an important role in combating the second wave. In conclusion, analysis based on table data may provide timely information about the dynamic progression of a pandemic and the long-term influence of an individual measure in the sequence on pandemic outcomes.

**Supplementary Information:**

The online version contains supplementary material available at 10.1186/s12982-022-00119-6.

## Introduction

Since the World Health Organization (WHO) declared coronavirus disease 2019 (COVID-19) a pandemic on 11 March 2020, countries around the globe have adopted different strategies to combat the pandemic. The progression of a pandemic is a complex stochastic process, in which a sequence of measures are implemented and pandemic outcomes occur between measures. Here, the pandemic outcomes include COVID-19 incidence, COVID-19 related admission to hospital or intensive care, COVID-19 death, general death, or economic indicators such as unemployment. The sequence of measures may be, for instance, a sequence of vaccine doses or a sequence of administrative interventions.

The pandemic progression is dynamic in the sense that the pandemic outcomes are results from the earlier measures and reasons for the subsequent measures and outcomes. Due to the dynamics of pandemic progression, a challenge arises in analysing the long-term influence of an individual measure in the sequence on pandemic outcomes. Probably the most dynamic progression occurred during the first wave of the pandemic, which completed a cycle of rising, plateau, and decline and base for public health outcomes such as COVID-19 deaths. The Nordic countries (Sweden, Denmark, Norway, and Finland) followed nearly the same time line of the progression during the first wave between March 2020 and August 2020, so we focus on the Nordic countries.

During the first wave, Sweden was representative of those strategies, emphasizing the mitigation of transmission and taking stepwise mild measures [1–3]. On the other hand, the other Nordic countries, i.e., Denmark, Finland, and Norway, were representative of the common strategies, emphasizing the suppression of transmission and taking invasive measures [3–6]. In the initial period, public health outcomes in Sweden were far poorer than the other Nordic countries but were gradually improved in the later period, i.e., curves were flattened, leading to a sense of optimism. Without considering the dynamics of the pandemic progression and the long-term influence of the early measure, Sweden continued with the mild measure recommendation upon arrival of the second wave, leading to a surge of poor public health outcomes. For instance, the COVID-19 mortality per 100,000 individuals in Sweden versus the other Nordic countries was 69.71 versus 13.40 between September 2020 and February 2021 (the second wave) [7–12].

There have been a large variety of analyses comparing the Nordic countries for the effectiveness of their strategies in combating COVID-19. One class of analyses is descriptive, which uses the daily or weekly count of public health and economic outcomes without adjustment for characteristic differences and updating pandemic situations [3–6]. Another class of analyses is statistical, which allows for adjustment of the characteristic differences and updating pandemic situations [13–15]. There are also mathematical analyses [16, 17] and political and cultural analyses [18–20]. However, few analyses involve the dynamics of pandemic progression and the long-term influences of individual measures in the sequence.

In this article, we demonstrate the dynamic progression of a pandemic and the long-term influence of an individual measure on pandemic outcomes. The pandemic outcomes include COVID-19 mortality, general mortality, COVID-19 incidence, and unemployment. The influence is described by the causal effect, which is defined in this article as an increase in the summary outcomes under different sequences of the Swedish and common measures. Furthermore, table data is used to demonstrate that analyses may provide timely information about the dynamic progression of a pandemic.

## Causal effects of Swedish strategy relative to common strategy on public health outcomes

### Dynamic progression of the pandemic

Here, we study the public health outcomes of COVID-19 mortality, general mortality, and COVID-19 incidence. We follow the population in Scandinavian countries during weeks 1–35, 2020. Please note that week 1, 2020 corresponds to the dates from 30 December 2019 to 5 January 2020.

The initial period of the pandemic took place around weeks 10–18 in the Nordic countries. Weeks 10–35 completed a cycle of rising, plateau, and decline and base for the public health outcome, and they are considered as the first wave of the pandemic [2, 3]. Because it is impossible to know when measures became effective, we divide the entire follow-up into four periods of approximately equal length: weeks 1–9, 10–18, 19–26, and 27–35. Let period $t$ $(t=1, 2, 3)$ indicate the three periods during weeks 10–35: period $1$ for weeks 10–18, period $2$ for weeks 19–26, and period $3$ for weeks 27–35. Please note that period 2 is one week shorter than periods 1 and 3. In Supplementary Information, we conduct a sensitivity analysis to show that when alternatively dividing the entire follow-up into weeks 1–9, 10–17, 18–26, and 27–35, the result only differs slightly, and the conclusion is the same. To examine the sensitivity of our methodology to periodization, we divide the follow-up into periods of different lengths and obtain essentially the same result and conclusion (results not shown).

During weeks 1–9, the pandemic had not yet broken out, so no measure was taken, and there was only outcome ${y}_{0}$ for general mortality in population ${p}_{0}$. During period $1$ (weeks 10–18), the exposure was ${z}_{1}=1$ for Swedish measure or ${z}_{1}=0$ for common measure and yielded outcome ${y}_{1}$ for population ${p}_{1}$. From here and on, the common measures refer to those adopted by the other Nordic countries. During period $2$ (weeks 19–26), the exposure was ${z}_{2}=1$ for Swedish measure or ${z}_{2}=0$ for common measure and yielded outcome ${y}_{2}$ for population ${p}_{2}$. During period $3$ (weeks 27–35), the exposure was ${z}_{3}=1$ for Swedish measure or ${z}_{3}=0$ for common measure and yielded outcome ${y}_{3}$ for population${p}_{3}$.

Outcome ${y}_{0}$ represents the initial health status and has an influence on outcomes ${y}_{1}$, ${y}_{2}$ and ${y}_{3}$. Thus, it is a stationary covariate and may confound the causal effects of exposures ${z}_{1}$, ${z}_{2}$ and ${z}_{3}$. Outcome ${y}_{1}$ represents the updating health status and pandemic situation during exposure ${z}_{1}$ and has an influence on outcomes ${y}_{2}$ and ${y}_{3}$. Thus, it is also a time-dependent covariate between exposures ${z}_{1}$ and ${z}_{2}$ and may confound the causal effects of exposures ${z}_{2}$ and ${z}_{3}$. Outcome ${y}_{2}$ represents the updating health status and pandemic situation during exposure ${z}_{2}$ and has an influence on outcome ${y}_{3}$. Thus, it is also a time-dependent covariate between exposures ${z}_{2}$ and ${z}_{3}$ and may confound the causal effect of exposure ${z}_{3}$.

### Confounding adjustment and the assumption of no hidden confounding covariates

Scandinavian countries are similar to one another in terms of economy, culture, and society. So, most of the stationary covariates, such as gender, education, and socioeconomic status, have similar distributions among these countries and thus do not confound the effects of exposures ${z}_{1}$, ${z}_{2}$ and ${z}_{3}$. As a result, there is no need to adjust for these covariates as is common practice in causal inference. Table 1 lists some characteristics of the populations in the Nordic countries. As seen in this table, the initial general mortality ${y}_{0}$ and population density $x$ differ considerably in different regions of these countries and may confound the causal effects. Therefore, we divide Sweden into six regions: Stockholm, Skåne, Gothenburg, Halland, Västmanland, and the rest of Sweden. Because COVID-19 mortality is low in Denmark, Finland, and Norway, we do not divide these countries into small regions. For the COVID-19 incidence, we divide Sweden into only two regions (Stockholm and the rest of Sweden) due to the data quality for the number of tested people for weeks 10–22.

**Table 1:** Characteristics of study populations in regions of the Nordic countries before the breakout of COVID-19: (1) Stockholm, (2) Skåne, (3) Göteborg, (4) Halland, (5) Västmanland, (6) the rest of Sweden, (1–6) Sweden as a whole, (7) Denmark, (8) Norway, (9) Finland

Characteristics	Populations in regions									
	(1)	(2)	(3)	(4)	(5)	(6)	(1–6)	(7)	(8)	(9)
Population size^a^,${n 10}^{3}$	2377	1378	1726	334	276	4237	10,328	5828	5328.2	5525.3
Sex^a^, ${n 10}^{3}$ (%)										
Male	1190 (50.06)	688 (49.93)	870 (50.41)	168 (50.30)	139 (50.36)	2142 (50.54)	5196 (50.31)	2899 (49.75)	2685 (50.39)	2728 (49.38)
Female	1187 (49.94)	690 (50.07)	856 (49.59)	166 (49.70)	137 (49.64)	2096 (49.46)	5132 (49.69)	2928 (50.25)	2643 (49.61)	2797 (50.62)
Age group^a^, n ${10}^{3} (\%)$										
0–19 years,	571 (24.03)	327 (23.73)	398 (23.06)	80 (23.95)	64 (23.19)	964 (22.75)	2404 (23.28)	1298 (22.28)	1255 (23.55)	1168 (21.14)
20–64 years	1426 (60.02)	781 (56.68)	992 (57.47)	181 (54.19)	152 (55.07)	2326(54.90)	5858 (56.72)	3377 (57.95)	3154 (59.20)	3126 (56.58)
65–years	379 (15.95)	270 (19.59)	336 (19.47)	73 (21.86)	60 (21.74)	947 (22.35)	2065 (20.00)	1152 (19.77)	919 (17.25)	1231 (22.28)
Population density^a^, $n$ per sq. km	365	128	73	62	54	11	25	137	15	18
General mortality rate^b^, $n$ per 100,000 person weeks	13.8	17.6	18.0	17.5	19.5	20.4	18.0	18.4	15.9	19.4
Unemployment rate^c^, %	7.0	11.1	7.0	6.3	9.0	8.6	7,6	5.4	3.8	7.7

Over a long period of time, all four countries are similar in terms of social and economic systems, social welfare systems including public health policies, education systems, and cultural traditions. Due to slightly different categorization of these social characteristics among these countries, their statistics are not listed here. Interested readers are referred to official statistics available on the webpages of Statistics Sweden, Statistics Denmark, Statistics Norway, and Statistics Finland

^a^Values based on December 2019

^b^Values based on weeks 1–9, 2020

^c^Values based on quarter 1, 2020

There may exist other potential confounding covariates, such as immigration status. Because different definitions of immigration status are used in these countries, it is difficult to adjust for immigration status without individual-level data. However, such covariates are often highly associated with population density, and as an approximation, we consider only population density as the confounding covariate in addition to the time-dependent outcomes ${y}_{0}, { y}_{1},$ and ${y}_{2}$. A summary of population densities, exposures, outcomes (covariates) and populations is given in Table 2 together with the probability models for the outcomes.

**Table 2:** A summary of population densities, exposures, outcomes, and the populations during different periods of the first wave

Period	Population density (Persons per sq. km)	Exposure: 1 for the Swedish measure 0 for the common measure	Outcome: COVID-19 mortality General mortality COVID-19 incidence	Population
Weeks 1–9	$x$	None	${y}_{0}$	${p}_{0}$
Weeks 10–18 (Period 1)	$x$	${z}_{1}=1$ or ${z}_{1}=0$	${y}_{1}$	${p}_{1}$
Weeks 19–26 (Period 2)	$x$	${z}_{2}=1$ or ${z}_{2}=0$	${y}_{2}$	${p}_{2}$
Weeks 27–35 (Period 3)	$x$	${z}_{3}=1$ or ${z}_{3}=0$	${y}_{3}$	${p}_{3}$

The outcome can also be a covariate for the subsequent exposures. The characteristics of study populations are described in Table 1. The study period is weeks 1–35, 2020

COVID-19 mortality and general mortality are measured as the number of deaths. They follow the Poisson distribution conditional on the history of previous exposures and covariates. The population is the amount of person weeks

COVID-19 incidence is measured as the number of cases. It follows the binomial distribution conditional on the history of previous exposures and covariates. The population is the number of tested persons

To summarize the confounding situation in the pandemic progression, we have the assumption of no hidden confounding covariates: (a) conditional on population density $x$ and outcome ${y}_{0}$, no other covariates confound the causal effect of an exposure sequence ${(z}_{1}, {z}_{2},{z}_{3})$; (b) conditional on population density $x$ and outcome ${y}_{1}$, no other covariates confound the causal effect of an exposure sequence ${(z}_{2},{z}_{3})$; (c) conditional on population density $x$ and outcome ${y}_{2}$, no other covariates confound the causal effect of exposure ${z}_{3}$. The assumption implies that to study the causal effects of exposures, we need to compare the outcomes of the exposures on the same level of population density and the most recent outcome prior to these exposures. In the Discussion section, we will discuss the limitation of our analysis linked to this assumption. In the Data sources section, we will describe the table data used for our analysis in detail.

### Analytic strategy

We will estimate two types of causal effects of the Swedish measures relative to the common measures: sequential causal effects and long-term causal effects. The sequential causal effect compares Swedish sequence versus common sequence for a summary outcome, for instance, Swedish sequence ${(z}_{1}, {z}_{2},{z}_{3})=(1, 1, 1)$ versus common sequence $(0, 0, 0)$ for summary outcome ${y}_{1}+{y}_{2}+{y}_{3}.$ Both the exposure sequences and the summary outcomes are observed for these causal effects, so we can apply regression to estimate them.

The long-term causal effect compares, for instance, mixed sequence ${(z}_{1}, {z}_{2},{z}_{3})=(1, 0, 0)$ to common sequence $(0, 0, 0)$ for the summary outcome ${y}_{1}+{y}_{2}+{y}_{3}$. Because mixed sequence cannot be observed, we cannot apply regression to estimate the long-term causal effect. Due to Robins [21], sequential causal inference is developed to estimate long-term causal effects under unobserved sequences of exposures by using observed data. Notably, the new general formula (G-formula) reveals a rather intuitive observation that the causal effect of an exposure sequence must be the sum of contributions of individual exposures in the sequence [22]. The new G-formula allows us to estimate the long-term causal effect from the estimated sequential causal effect without introducing additional modeling assumptions. In the following subsections, we will describe analyses and the results in detail.

### Sequential causal effects of the Swedish sequences relative to common sequences

We estimate the following three sequential causal effects of interest: (i) an increase in summary outcome ${y}_{1}+{y}_{2}+{y}_{3}$ during periods 1, 2, and 3 (weeks 10–35) under the Swedish sequence ${(z}_{1}, {z}_{2}, {z}_{3})=(1, 1, 1)$ relative to the common sequence $(0, 0, 0)$, (ii) an increase in summary outcome ${y}_{2}+{y}_{3}$ during periods 2 and 3 (weeks 19–35) under the Swedish sequence $({z}_{2},{z}_{3})=(1, 1)$ relative to the common sequence $(0, 0)$, and (iii) an increase in outcome ${y}_{3}$ during period 3 (week 27–35) under the Swedish measure ${z}_{3}=1$ relative to the common measure $0$. In the context of the pandemic, the exposure sequence takes either the Swedish sequence or the common sequence. The outcomes are observed under the exposure sequences in causal effects (i), (ii) and (iii), so we can use regression to estimate these causal effects [21, 22]. The results are summarized in Table 3. A detailed description of the probability models and regression models is given in the Method section below.

**Table 3:** Estimate, 95% CI, and p-value for sequential causal effects of the Swedish sequence relative to the common sequence on summary public health outcomes

$$\left(\begin{array}{c}\mathrm{Estimate}\\ 95\mathrm{\% CI}\\ \text{p-}\mathrm{value}\end{array}\right)$$ for sequential causal effect on public health outcome			
Outcome	COVID-19 mortality	General mortality	COVID-19 incidence
Causal effect			
(i)	42.6, (41.0, 44.1) < 0.001	25.0 (18.7, 30.7) < 0.001	19,094.5 (18,916.6, 19,212.3) < 0.001
$\left(\mathrm{ii}\right)$	17.5 (15.7, 19.3) < 0.001	$-$ 20.0 ($-$ 28.2, $-$ 11.1) < 0.001	8642.4 (6776.9, 10,507.8) < 0.001
(iii)	1.9 (0.5, 3.3) 0.01	$-$ 17.6 ($-$ 22.5, $-$ 12.6) < 0.001	3120.0 (2927.2, 3312.9) < 0.001

The causal effects are

(i) An increase in summary outcome ${y}_{1}+{y}_{2}+{y}_{3}$ per 100,000 individuals during weeks 10–35 (periods 1, 2, and 3) under the Swedish sequence ${(z}_{1}, {z}_{2},{z}_{3})=(1, 1, 1)$ relative to the common sequence ${(z}_{1}, {z}_{2},{z}_{3})=(0, 0, 0)$

(ii) An increase in summary outcome ${y}_{2}+{y}_{3}$ per 100,000 individuals during weeks 19–35 (periods 2 and 3) under the Swedish sequence $({z}_{2},{z}_{3})=(1, 1)$ relative to the common sequence $({z}_{2},{z}_{3})=(0, 0)$

(iii) An increase in outcome ${y}_{3}$ per 100,000 individuals during weeks 27–35 (periods 3) under the Swedish measure ${z}_{3}=1$ relative to the common measure ${z}_{3}=0$

As shown from causal effect (i) in Table 3, the Swedish strategy performed far worse than the common strategy throughout the complete follow-up (weeks 10–35) for all public health outcomes: it led, per 100,000 individuals, to 42.6 (95% Confidence Interval: 41.0–44.1) more COVID-19 deaths, 25.0 (18.7–30.7) more general deaths and 19,094.5 (18,916.6–19,212.3) more COVID-19 incidences. As shown from causal effects (ii) and (iii), the Swedish strategy improved its performance during weeks 19–35 and 27–35, particularly for general mortality: it led, per 100,000 individuals, to 20.0 (11.1–28.2) fewer general deaths during week 19–35 and 17.6 (12.6–22.5) fewer general deaths during week 27–35. The reason might be that the Swedish public health system regained its usual level of general medical care after the early pandemic period of weeks 10–18. In the Supplementary Information, we conduct a sensitivity analysis to show that the improvement was not due to population change caused by more general deaths during weeks 10–18.

### Long-term causal effects of the Swedish measures relative to common measures

To reveal the critical role of the early measures in combating the pandemic, we then estimate two long-term causal effects (iv) and (v). Causal effect (iv) is an increase in summary outcome ${y}_{1}+{y}_{2}+{y}_{3}$ during periods 1, 2, and 3 (weeks 10–35) under the mixed sequence ${(z}_{1}, {z}_{2},{z}_{3})=(1, 0, 0)$ relative to the common sequence $({0}, 0, 0)$, and it describes the long-term influence of the Swedish measure during period 1 on the summary outcome during periods 1, 2, and 3. Causal effect (v) is an increase in summary outcome ${y}_{2}+{y}_{3}$ during periods 2 and 3 (weeks 19–35) under the mixed sequence $({z}_{2},{z}_{3})=(1, 0)$ relative to the common sequence $(0, 0)$, and it describes the long-term influence of the Swedish measure during period 2 on the summary outcome during periods 2 and 3.

Here, the outcomes are not observable because the population is never exposed to mixed sequence ${(z}_{1}, {z}_{2},{z}_{3})=(1, 0, 0)$ or $({z}_{2},{z}_{3})=(1, 0)$, so we cannot use regression to estimate long-term causal effects (iv) and (v). However, by the new G-formula [22], sequential causal effect is a sum of contributions from individual exposures in the sequence, and therefore we obtain the equality that causal effect (ii) is equal to the sum of causal effects (v) and (iii). The equality is illustrated by the fact that the sequences in causal effects (ii), (v) and (iii) are $({z}_{2},{z}_{3})=(1, 1)$, $({z}_{2},{z}_{3})=(1, 0)$ and ${z}_{3}=1$. By using this equality, we obtain the estimate of causal effect (v) from the estimates of causal effects (ii) and (iii). Similarly, causal effect (i) is equal to the sum of causal effects (iv), (v), and (iii). We obtain the estimate of causal effect (iv) from the estimates of causal effects (i), (v) and (iii). A detailed description of this method is given in the Method section below. The estimates of causal effects (iv) and (v) are presented in Table 4.

**Table 4:** Estimate, 95% CI, and p-value for long-term causal effects of the Swedish measure relative to the common measure on public health outcome during different periods

$$\left(\begin{array}{c}\mathrm{Estimate}\\ 95\mathrm{\% CI}\\ \text{p-}\mathrm{value}\end{array}\right)$$ for long-term causal effect on public health outcome			
Outcome			
Causal effect	COVID-19 mortality	General mortality	COVID-19 incidence
(iv)	25.1 (23.0, 27.0) < 0.001	44.3 (34.5, 54.2) < 0.001	10,422.1 (8553.8, 12,290.5) < 0.001
(v)	15.6 (13.3,18.0) < 0.001	$-$ 2.1 ($-$ 12.2, 8.0) 0.7	5522.3 (3661.0, 7383.6) < 0.001

The causal effects are

(iv) An increase in summary outcome ${y}_{1}+{y}_{2}+{y}_{3}$ per 100,000 individuals during weeks 10–35

(periods 1, 2, and 3) under the mixed sequence ${(z}_{1}, {z}_{2},{z}_{3})=(1, 0, 0)$ relative to the common

sequence $${(z}_{1}, {z}_{2},{z}_{3})=({0},0, 0)$$

(v) An increase in summary outcome ${y}_{2}+{y}_{3}$ per 100,000 individuals during weeks 19–35

(periods 2 and 3) under the mixed sequence $({z}_{2},{z}_{3})=(1, 0)$ relative to the common sequence

$$({z}_{2},{z}_{3})=(0, 0)$$

Table 4 shows that the early Swedish measure had a long-term and significant influence on public health outcomes. As shown from causal effects (iv), the Swedish measure during the early period (weeks 10–18) led, per 100,000 individuals, to 25.1 (23.0–27.0) more COVID-19 deaths, 44.3 (34.5–54.2) more general deaths and 10,422.1 (8553.8–12,290.5) more COVID-19 incidences for the whole first wave (weeks 10–35). From causal effects (iv), (v) in Table 4, and (iii) in Table 3 together, we see a continual improvement in the Swedish measures relative to the common measures along weeks 10–18, 19–26, and 27–35.

## Causal effects of the Swedish strategy relative common strategy on unemployment

Here, we study the economic outcome of unemployment in an analogy to the public health outcomes. We divide the complete follow-up (quarters 1–3) into three periods: quarters 1, 2, and 3. During quarter 1, no measures were taken, and even if some measures had been taken, they would not have influenced unemployment in the current quarter, so there is only unemployment ${y}_{1}$ from labour force ${p}_{1}$ in quarter 1. During quarter 2, the exposure is ${z}_{2}=1$ for Swedish measure or ${z}_{2}=0$ for common measure, yielding unemployment ${y}_{2}$ in labour force ${p}_{2}$. During quarter 3, the exposure is ${z}_{3}=1$ for Swedish measure or ${z}_{3}=0$ for common measure, yielding unemployment ${y}_{3}$ in labour force ${p}_{3}$.

To adjust for confounding, we have the following assumption of no hidden confounding covariates: (a) conditional on population density $x$ and outcome ${y}_{1}$, no other covariates confound the causal effect of exposure sequence ${(z}_{2},{z}_{3})$; (b) conditional on population density $x$ and outcome ${y}_{2}$, no other covariates confound the causal effect of exposure ${z}_{3}$. With the assumption and the data, we will estimate the following three causal effects for unemployment: (i) an increase in summary unemployment ${y}_{2}+{y}_{3}$ during quarters 2–3 under the Swedish sequence $({z}_{2},{z}_{3})=(1, 1)$ relative to the common sequence $(0, 0)$, (ii) an increase in unemployment ${y}_{3}$ during quarter 3 under the Swedish measure ${z}_{3}=1$ relative to the common measure $0$, (iii) an increase in summary unemployment ${y}_{2}+{y}_{3}$ during quarters 2–3 under the mixed sequence $({z}_{2},{z}_{3})=(1, 0)$ relative to the comment sequence $(0, 0)$. Causal effects (i) and (ii) are sequential. Causal effect (iii) is long-term. In the Method section, we describe the probability model and the regression model in detail. The estimates of causal effects (i), (ii), and (iii) are presented in Table 5.

**Table 5:** Estimate, 95% CI, and p-value for causal effects of the Swedish strategy relative to the common strategy on unemployment during different periods

$$\left(\begin{array}{c}\mathrm{Estimate}\\ 95\mathrm{\% CI}\\ \text{p-} \mathrm{value}\end{array}\right)$$ for causal effect on unemployment	
Outcome	Unemployment
Causal effect	
(i)	1177.0 (1088.8, 1265.1) < 0.001
$(\mathrm{ii})$	528.4 (480.2, 576.5) < 0.001
$(\mathrm{iii})$	648.6 (555.7, 751.5) < 0.001

The causal effects are

(i) An increase in summary unemployment ${y}_{2}+{y}_{3}$ per 100,000 individuals during quarters 2 and 3 under the Swedish sequence $({z}_{2},{z}_{3})=(1, 1)$ relative to the comment sequence $({z}_{2},{z}_{3})=(0, 0)$. It is a sequential causal effect

(ii) An increase in unemployment ${y}_{3}$ per 100,000 individuals during quarter 3 under the Swedish measure ${z}_{3}=1$ relative to the common measure ${z}_{3}=0$. It is a sequential causal effect

(iii) An increase in summary unemployment ${y}_{2}+{y}_{3}$ per 100,000 individuals during quarters 2 and 3 under the mixed sequence $({z}_{2},{z}_{3})=(1, 0)$ relative to the comment sequence $({z}_{2},{z}_{3})=(0, 0)$. It is a long-term causal effect

As shown from causal effects (i) and (ii) in Table 5, the Swedish strategy performed worse than the common strategy during quarters 2–3 and quarter 3 for unemployment: it led, per 100,000 individuals, to 1177.0 (1088.8–1265.1) more unemployment during quarters 2–3 and 528.4 (480.2–576.5) more unemployment during quarter 3. As shown from causal effect (iii), the early measures during quarter 2 had a certain long-term mitigating influence on unemployment, yielding a mild increase of 648.6 (555.7–751.5) unemployment per 100,000 individuals during quarters 2–3.

## Discussion

This article analyses the dynamic progression of the first wave in the Nordic countries and has two major findings. First, the early mild measure had a long-term and significant influence on public health outcomes. Second, the early mild measure led to a certain degree of long-term mitigating influence on unemployment. The article demonstrates that the long-term influence of an individual measure in the sequence can be sizable and significant and may play an important role in combating COVID-19 or the future pandemic.

The analysis in this article contributes to combating the pandemic in two aspects. First, to the best of our knowledge, the dynamics of pandemic progression has not been sufficiently studied. Our analysis demonstrates that the long-term influence of individual measure in the sequence can be estimated in the framework of sequential causal inference [21, 22]. Second, the data used for our analysis is the same table data as used for descriptive analyses. This implies that our analysis can be conducted at the same time as descriptive analysis. We believe that, by the same method, one can analyse the dynamic progression of pandemic under vaccination, where the exposure sequence can be a sequence of vaccine doses, the outcome can be COVID-19 incidence, admission to hospital or intensive care, or COVID-19 death, the study population can be one nation, and the data can be table data recording the frequencies of vaccine sequences and outcomes over the time. The causal effect of individual dose over prolonged period has been one of the major issues in combating the current COVID-19 [23].

Though our analyses based on table data can be timely, they have several limitations in comparison to individual-level data. First, some of the covariates are individual based, such as income and education, and it is impossible to assess their confounding influence on the causal effect without individual-level data. Second, it is difficult to conduct quality control of pandemic outcomes. In this article, we use COVID-19 incidences among tested people during different periods to study the transmission. Ideally, we might use admission to inpatient care and intensive care as the outcome. However, different countries had different policy for admission; for instance, Denmark had a much higher admission rate than Sweden, so it would be problematic to use admission as an outcome of the transmission. The second limitation exists for both table data and individual-level data.

## Data sources

As recommended by the WTO, all four Nordic countries identify COVID-19 death as death for which a positive COVID-19 PCR test was recorded within 30 days. Unemployment is measured as the number of unemployed persons aged 15–74, and employment as the number of employed persons aged 15–74. These numbers are produced by the labour force surveys conducted in individual countries following the European Union Council Regulation. The labour force is the sum of employed and unemployed persons. Population density is measured as the number of inhabitants per square kilometre.

The Public Health Agency of Sweden and the National Board of Health and Welfare are two national agencies accountable to the Swedish government. The Public Health Agency has an overall responsibility for the control of communicable diseases, such as COVID-19. From its public webpage (https://www.folkhalsomyndigheten.se/the-public-health-agency-of-sweden/), we obtained the number of tested people and COVID-19 incidences in different regions of Sweden. The National Board of Health and Welfare has a general responsibility for social welfare and healthcare including knowledge support and statistics. From its public website (https://www.government.se/government-agencies/national-board-of-health-and-welfare--socialstyrelsen/), we obtained the COVID-19 mortality and general mortality. Statistics Sweden is a government agency that produces official statistics. From its public website (https://www.scb.se/en), we obtained the population size, population density, unemployment, and labour force.

The Danish Health Authority is the national agency for health care. Statistics Denmark is the national agency that produces official statistics. All table data relevant to our article were obtained from the public site of Statistics Denmark (https://www.dst.dk/en).

The Finnish Institute for Health and Welfare is the national agency for healthcare and welfare in Finland. From its public website (https://thl.fi/en/web/thlfi-en), we obtained the numbers of tested people and COVID-19 incidences and COVID-19 mortality. Statistics Finland is the national agency that produces official statistics. From its public website (https://www.stat.fi/index_en.html), we obtained general mortality, population size, population density, unemployment, and labour force.

The Norwegian Institute of Public Health is the national agency for public healthcare in Norway. From its public website (https://www.fhi.no/en/), we obtained the numbers of tested people and COVID-19 incidences, COVID-19 mortality, and general mortality. Statistics Norway is the national agency that produces official statistics. From its public website (https://www.ssb.no/en), we obtained population size, population density, unemployment, and labour force. In the Supplementary Information, we provide table data relevant to this article.

## Method

### Method of estimating causal effects on public health outcomes

Here, we estimate the causal effect of the Swedish strategy relative to the common strategy on COVID-19 mortality, general mortality, and COVID-19 incidence. COVID-19 mortality and general mortality are measured as the number of deaths during a follow-up of person weeks in the population. They follow the Poisson distribution conditional on the history of previous exposures and covariates. COVID-19 incidence is measured as the number of cases among a group of tested persons. It follows the binomial distribution conditional on the history of previous exposures and covariates. The regression models are given in detail below.

Causal effect (i) is an increase in summary outcome $s={y}_{1}+{y}_{2}+{y}_{3}$ during periods 1, 2, and 3 under the Swedish sequence ${(z}_{1}, {z}_{2},{z}_{3})=(1, 1, 1)$ relative to the common sequence ${(z}_{1}, {z}_{2},{z}_{3})=(0, 0, 0)$. Let ${r}_{0}={y}_{0}/{p}_{0}$, which is the mortality rate during weeks 1–9. Let $w$ be the variable that describes the exposure sequence during periods 1, 2, and 3 such that $w=1$ for the Swedish sequence $(1, 1, 1)$ or 0 for the common sequence $(0, 0, 0)$. The regression model for the expectation of the summary outcome $s={y}_{1}+{y}_{2}+{y}_{3}$ is

$$E\left(s | x,{r}_{0}, w\right)=({p}_{1}+{p}_{2}+{p}_{3})\left(\alpha +\gamma x+{\delta r}_{0}+\beta w\right).$$

Here, the link function is identity function; the covariates are density $x$ and mortality rate ${r}_{0}={y}_{0}/{p}_{0}$ during weeks 1–9; the exposure is $w$ (Swedish or common sequence); the amount ${p}_{t} (t=1, 2, 3)$ of person weeks during period $t$ is fixed as a constant. We use linear model with only the main effect of exposure $w$ for the following reasons. First, the exact functional form for the nuisance variables $x$ and ${r}_{0}$ is unknown, and a reasonable assumption is linear form. Second, by sensitivity analysis, the effect modification of the main effect of exposure $w$ by $x$ and ${r}_{0}$ is small. Under the assumption of no hidden confounding covariates, we have that

$$\text{causal\, effect (i)}=E\left(s | x,{r}_{0}, w=1\right)-E\left(s | x,{r}_{0 }, w=0\right)=\left({p}_{1}+{p}_{2}+{p}_{3}\right)\beta .$$

From $\beta$, we obtain causal effect (i) under the assumption of no hidden confounding covariates.

Causal effect (ii) is an increase in summary outcome ${y}_{2}+{y}_{3}$ during periods 2 and 3 under the Swedish sequence $({z}_{2},{z}_{3})=(1, 1)$ relative to the common sequence $(0, 0)$. Using the same method as for causal effect (i), we obtain the regression model for the expectation of the summary outcome ${y}_{2}+{y}_{3}$ to estimate the causal effect (ii). In the model, the link function is identity function; the covariates are density $x$ and rate ${r}_{1}={y}_{1}/{p}_{1}$ during period 1; the exposure is the variable that describes the exposure sequence during periods 2 and 3 (Swedish or common sequence); the amount ${p}_{t} (t=1, 2, 3)$ of person weeks during period $t$ is fixed as a constant.

Causal effect (iii) is an increase in outcome ${y}_{3}$ during period 3 under the Swedish measure ${z}_{3}=1$ relative to the common measure $0$. Similarly, we obtain the regression model for the expectation of outcome ${y}_{3}$ to estimate the causal effect (iii). Here, the link function is identity function; the covariates are density $x$ and rate ${r}_{2}={y}_{2}/{p}_{2}$ during period 2; the exposure is ${z}_{3}$; the amount ${p}_{2}$ of person weeks during period $2$ and ${p}_{3}$ during period $3$ are fixed as constants.

Causal effect (iv) is an increase in summary outcome ${y}_{1}+{y}_{2}+{y}_{3}$ during periods 1, 2, and 3 under the mixed sequence $({z}_{1}, {z}_{2},{z}_{3})=(1, 0, 0)$ relative to the common sequence ${(z}_{1}, {z}_{2},{z}_{3})=(0, 0, 0)$. Here, the exposures ${z}_{2}, {z}_{3}$ in the mixed sequence are set at 0, namely, common measures, so this causal effect describes the long-term influence of the Swedish measure ${z}_{1}=1$ during period 1 on the summary outcome throughout periods 1, 2, and 3. Causal effect (v) is an increase in the summary outcome ${y}_{2}+{y}_{3}$ during weeks 19–35 under the mixed sequence $( {z}_{2},{z}_{3})=(1, 0)$ relative to the common sequence $({z}_{2},{z}_{3})=(0, 0)$. It describes long-term influence of the Swedish measure ${z}_{2}=1$ during period 2 on the summary outcome throughout periods 2 and 3.

The mixed sequences in causal effects (iv) and (v) were never exposed to the population, so their outcomes were not observed, and these causal effects cannot be estimated by regressions. On the other hand, by applying Theorems 1 and 2 of Wang and Yin[22], we obtain the equality that

$$\text{causal effect (ii) } =\, \text{causal effect (v) }+\text{causal effect (iii).}$$

The﻿ equality reveals a rather intuitive observation that sequential causal effect is a sum of the contributions from individual exposures in the sequence. Please note that the exposure sequences in causal effects (ii), (v) and (iii) are $({z}_{2},{z}_{3})=(1, 1)$, $({z}_{2},{z}_{3})=(1, 0)$ and ${z}_{3}=1$ respectively. Based on this equality, the estimate of causal effect (v) is obtained by using the obtained estimates of causal effects (ii) and (iii). Similarly, by applying Theorems 1 and 2 of Wang and Yin [22], we obtain the equality that

$$\text{causal effect (i) }=\text{causal effect (iv) }+\text{causal effect (v) + causal effect (iii).}$$

Please﻿ note that the exposure sequences in causal effects (i), (iv), (v) and (iii) are $({z}_{1},{z}_{2},{z}_{3})=(1, 1, 1)$, $({z}_{1},{z}_{2},{z}_{3})=(1, 0, 0)$, $({z}_{2},{z}_{3})=(1, 0)$ and ${z}_{3}=1$, respectively. Based on this equality, the estimate of causal effect (iv) can be obtained by using the obtained estimates of causal effects (i), (v) and (iii).

The confidence interval and p-values of causal effects (i)-(v) are obtained using Monte Carlo simulation based on the probability models and the obtained regression models.

### Method of estimating causal effects on unemployment

Here, we estimate the causal effect of the Swedish strategy relative to the common strategy adopted by other Nordic countries on unemployment. Unemployment is measured as the number of unemployed persons among labour force (the sum of employed and unemployed persons). Therefore, we assume that unemployment follows the binomial distribution. The regression models are described below.

Causal effect (i) is an increase in summary unemployment ${s=y}_{2}+{y}_{3}$ during quarters 2–3 under the Swedish sequence $( {z}_{2},{z}_{3})=(1, 1)$ relative to the common sequence $( {z}_{2},{z}_{3})=(0, 0)$. Let ${r}_{1}={y}_{1}/{p}_{1}$ be the unemployment rate during quarter 1. Denote the exposure by $w$, which takes 1 for the Swedish sequence or 0 for the common sequence. Then, the regression model for the expectation of ${s=y}_{2}+{y}_{3}$ is

$$E\left(s | x,{r}_{1}, w\right)=\left({p}_{2}+{p}_{3}\right)\left(\alpha +\gamma x+\delta {r}_{1}+\beta w\right).$$

Under﻿ the assumption of no hidden confounding covariates, we have

$$\mathrm{causal effect }\left(\mathrm{i}\right)=E\left(s | x,{r}_{1}, w=1\right)-E\left(s | x,{r}_{1}, w=0\right)=\left({p}_{2}+{p}_{3}\right)\beta .$$

From﻿ estimate of $\beta$, we obtain estimate of causal effect (i) under the assumption of no hidden confounding covariates. Similarly, we can estimate causal effect (ii) which is an increase in outcome ${y}_{3}$ under the Swedish measure ${z}_{3}=1$ relative to the common measure ${z}_{3}=0$ during quarter 3.

Causal effect (iii) is an increase in summary outcome ${y}_{2}+{y}_{3}$ under the mixed sequence $( {z}_{2},{z}_{3})=(1, 0)$ relative to the common sequence $( {z}_{2},{z}_{3})=(0, 0)$ during quarters 2–3. By applying Theorems 1 and 2 of Wang and Yin [22], we obtain the equality that

$$\text{causal effect (i) }=\text{causal effect (iii) }+\text{ causal effect (ii)}$$

Please﻿ note that the exposure sequences in causal effects (i), (iii) and (ii) are $({z}_{2},{z}_{3})=(1, 1)$, $({z}_{2},{z}_{3})=(1, 0)$ and ${z}_{3}=1$, respectively. With the equality, we have that causal effect (iii) is equal to

$$\text{causal effect (iii) = causal effect (i)}-\text{causal effect (ii)}$$

Therefore﻿, we obtain the estimate of causal effect (iii) from those of causal effects (i) and (ii). The confidence intervals and p-values of causal effects (i), (ii), and (iii) are obtained using Monte Carlo simulation based on the probability models and the obtained regression models.

## Supplementary Information

**Additional file 1.** 1) Sensitivity analysis for the impact of an alternative follow-up split on the estimation. 2) Sensitivity analysis for the impact of population change on the estimation. 3) Data and code, available in [Zenodo] at http://doi.org/10.5281/zenodo.5136641 [24].

## Data Availability

The data and material are publicly available and can be assessed as described in the Method section. A data set relevant to the analysis is given in the Supplementary Information together with the code producing the result.
