# Supplementary material for: Dynamics of COVID-19 progression and the long-term influences of measures on pandemic outcomes
Source: Emerg Themes Epidemiol. 2022 Dec 22;19:10. doi: 10.1186/s12982-022-00119-6 (PMC9773575; doi:10.1186/s12982-022-00119-6)
Supplement: Supplementary file 1 — Additional file 1. 1) Sensitivity analysis for the impact of an alternative follow-up split on the estimation. 2) Sensitivity analysis for the impact of population change on the estimation. 3) Data and code, available in [Zenodo] at http://doi.org/10.5281/zenodo.5136641 [24]. [file 12982_2022_119_MOESM1_ESM.docx]

Supplementary Information for

**Dynamics of COVID-19 progression and the long-term influences of measures on pandemic outcomes**

Yihong Lan, Li Yin and Xiaoqin Wang

**(1) Sensitivity analysis for the impact of an alternative follow-up split on the estimation.**

A summary of population densities, exposures, outcomes (covariates) and populations is given in Table 2. As described in the Method Section, we have the assumption of no hidden confounding covariates, i.e., given the population density and outcome in a period, no other covariates exist that confound the causal effects of exposures in the subsequent periods. With the assumption and the data, we estimate five causal effects of interest for COVID-19 mortality, general mortality and COVID-19 incidence. The five causal effects are described in Table 3 and Table 4 of the article. The regression models used in the estimation are described in detail in the Method section.

In the analysis for the result in Tables 3 and 4, we split the complete follow-up (weeks 1-35) into weeks 1-9, 10-18, 19-26, and 27-35. Here, instead we split the complete follow-up into weeks 1-9, 10-17, 18-26, and 27-35 and conducted the same analysis for COVID-19 mortality and general mortality. Due to the data quality for the number of tested people for weeks 10-22, we do not conduct sensitivity analysis for COVID-19 incidence.

The result is presented in Table S1. As shown in this table, the estimated causal effects are only slightly different for the two ways of splitting. Therefore, the conclusions are the same for the two ways of splitting.

**(2) Sensitivity analysis for the impact of population change on the estimation.**

When estimating the causal effects for the outcomes of COVID-19 mortality and general mortality, we need to calculate the person weeks $p_{0}$, $p_{1}$,$p_{2}$, and $p_{3}$, where weeks are the number of weeks during a period and persons are the population size during this period. We use the population size of December 2019 for all the estimations. This approximation is usually reasonable because general mortality is far smaller than the population size. On the other hand, there is a considerable increase in general deaths during the pandemic and thus a reduction in the population size. To examine the influence of population change, we conducted a sensitivity analysis, in which we subtracted the population size by the general mortality and then used the obtained population size to estimate the causal effect on general mortality. The result is presented in Table S2. As shown in this table, the estimated causal effects are nearly identical to those obtained with the population size of December 2019. Therefore, we conclude that population change has little impact on the estimation of the causal effect.

**(3) Data and code**

It is publicly available in [Zenodo] at http://doi.org/10.5281/zenodo.5136641.

| $\left( \begin{matrix} \mathrm{Estimate} \\ 95\% CI \\ \text{p-}\mathrm{value} \end{matrix} \right)$for causal effect estimated with two follow-up splits | | | | |
| --- | --- | --- | --- | --- |
| Causal effect | Split (a) | | Split (b) | |
|  | COVID-19 mortality | General mortality | COVID-19 mortality | General mortality |
| (i) | 42.6  (41.0, 44.1)  < 0.001 | 25.0  (18.7, 30.7)  < 0.001 | 42.6  (41.1, 44.2)  < 0.001 | 24.7  (18.7, 30.7)  < 0.001 |
| $\left( \mathrm{ii} \right)$ | 17.5,  (15.7, 19.3)  < 0.001 | $-$20.0  ($-$28.2, $-$11.1)  < 0.001 | 21.3  (19.4, 23.3)  < 0.001 | $-$16.7  ($-$26.1, $-$7.3)  < 0.001 |
| (iii) | 1.9  (0.5, 3.3)  0.01 | $-$17.6  ($-$22.5, $-$12.6)  < 0.001 | 2.0  (0.6, 3.4)  0.01 | $-$18.8  ($-$23.7, $-$13.9)  < 0.001 |
| (iv) | 25.1  (23.0, 27.0)  < 0.001 | 44.3  (34.5, 54.2)  < 0.001 | 21.3  (19.3, 23.3)  < 0.001 | 41.4  (30.7, 52.1)  < 0.001 |
| (v) | 15.6  (13.3,18.0)  < 0.001 | $-$2.1  ($-$12.2, 8.0)  0.7 | 19.4  (16.9,21.8)  < 0.001 | 2.1  ($-$8.8, 12.9)  0.7 |

Table S1. Sensitivity analysis for the impact of alternative follow-up split. Causal effects: (i)-(v) described in Table 3 and 4. Outcomes: COVID-19 mortality and general mortality. Two follow-up splits: (a) weeks 1-9, 10-18, 19-26, 27-35 (used in Tables 3 and 4) and (b) weeks 1-9, 10-17, 18-26, 27-35.

Table S2. Sensitivity analysis for impact of population change caused by general mortality. Causal effects: (i)-(v) described in Table 3 and 4. Outcome: general mortality. Two populations: (a) population of December 2019 (used in Tables 3 and 4) and (b) population (a) subtracted by general mortality in earlier periods.

| $\left( \begin{matrix} \mathrm{Estimate} \\ 95\% CI \\ \text{p-}\mathrm{value} \end{matrix} \right)$ for causal effect estimated with two populations | | |
| --- | --- | --- |
| Causal effect | Population (a) | Population (b) |
| (i) | 25.0  (18.7, 30.7)  < 0.001 | 24.7  (18.7, 30.8)  < 0.001 |
| $\left( \mathrm{ii} \right)$ | $-$20.0  ($-$28.2, $-$11.1)  < 0.001 | $-$19.7  ($-$28.3, $-$11.2)  < 0.001 |
| (iii) | $-$17.6  ($-$22.5, $-$12.6)  < 0.001 | $-$17.6  ($-$22.6, $-$12.6)  < 0.001 |
| (iv) | 44.3  (34.5, 54.2)  < 0.001 | 44.5  (34.6, 54.3)  < 0.001 |
| (v) | $-$2.1  ($-$12.2, 8.0)  0.7 | $-$2.1  ($-$12.2, 8.0)  0.7 |
